# Spatial differentiation and influencing factors of effective phosphorus in cultivated soil in the water source area of the mid-route of South-to-North water transfer project

**DOI:** 10.3389/fmicb.2024.1463291

**Published:** 2024-09-04

**Authors:** Zhengxiang Wu, Yang Zhou, Miao Wang

**Affiliations:** ^1^Key Laboratory of Natural Disaster and Remote Sensing of Henan Province, Nanyang Normal University, Nanyang, Henan, China; ^2^Rural Revitalization Institute, Nanyang Normal University, Nanyang, Henan, China; ^3^Nanyang Development Strategy Institute, Nanyang Normal University, Nanyang, Henan, China

**Keywords:** available phosphorus, cultivated soil, spatial differentiation, geostatistics, geodetectors

## Abstract

The long-term application of phosphate fertilizers in agricultural production leads to a large accumulation of phosphorus in the soil. When it exceeds a certain limit, phosphorus will migrate to surrounding water bodies through surface runoff and other mechanisms, potentially causing environmental risks such as eutrophication of water bodies and increasing the risk of water source pollution. This study takes Shiyan City, the water resources area of the mid-route of the South-to-North Diversion Project (MSDP), as the study area. Based on 701 sampling points of topsoil, geostatistics and geodetectors were used to explore the spatial heterogeneity and influencing factors of available phosphorus (AP) in the topsoil of the area. The results show that the effective phosphorus content in the topsoil of the study area ranges from 0.30 to 146.00 mg/kg, with an average value of 14.28 mg/kg, showing strong variability characteristics. Geostatistical analysis shows that among all theoretical models, the exponential model has the best fitting effect, with a lump gold effect of 0.447 and a range of 82,000 m. The soil available phosphorus content shows an increasing trend from the Central Valley lowlands to the surrounding mountainous hills. Among them, elevation is the main controlling factor for the spatial variation of available phosphorus in the topsoil, followed by soil types, planting systems, annual precipitation, and organic matter. The non-linear enhancement or dual-factor enhancement among various environmental factors reveals the diversity and complexity of spatial heterogeneity affecting available phosphorus content in cultivated soil. This study could provide scientific references for maintaining ecological security in the water source area of the MSDP, improving the precise management of AP, and enhancing cultivated land quality.

## 1 Introduction

Available phosphorus (AP) in cultivated soil is an important factor that characterizes the abundance and deficiency of soil phosphorus nutrition and quality of the environment (Liu et al., 2022; Zhang et al., 2021). As one of the three essential nutrients for plants, phosphorus plays an irreplaceable role in their life cycle. The lack of soil AP can limit crop growth and affect crop yield (Bieluczyk et al., 2024; Zicker et al., 2018). With the continuous improvement of land use intensity, the application of phosphorus fertilizers has become widespread in agricultural production to ensure the quality and yield of agricultural production. Compared to nitrogen and potassium, phosphorus fertilizer is easily adsorbed and converted into insoluble phosphate that is difficult for crops to absorb by the surface of soil particles or iron and aluminum oxides in the soil after being applied to the soil (Du et al., 2021). Therefore, the seasonal utilization rate of phosphorus fertilizer is low, ranging from 10 to 25% (Rowe et al., 2015). At the same time, farmers are accustomed to using phosphorus fertilizers that often exceed the actual phosphorus requirements of crops. Long-term fertilization leads to a large accumulation of phosphorus in the soil (Yang et al., 2017; Khan et al., 2018). Although phosphorus accumulation can improve the soil's phosphorus supply capacity to crops, when it exceeds a certain limit, phosphorus may migrate to surrounding water bodies through surface runoff and other mechanisms. This not only results in fertilizer wastage but also increases environmental risks, such as eutrophication of water bodies, posing significant threats to ecological health and the sustainable development of agriculture (Holger et al., 2018; Liu et al., 2016). Therefore, fully understanding the spatial layout characteristics of soil AP in regional farmland is crucial for optimizing farmland management measures, applying phosphorus fertilizers more effectively, and reducing phosphorus loss and non-point source pollution in water bodies.

The application of soil AP plays an important role in ensuring food yield increase and sustainable development of soil phosphorus fertility, which have attracted widespread attention from scholars both at home and abroad. Scholars have conducted extensive research on the spatial distribution characteristics of AP from the perspective of soil properties (Sattari et al., 2012), crop types (Lv et al., 2022), crop rotation systems (Chen et al., 2024; Lü et al., 2022), topography (Hua et al., 2020), and soil types (Bai et al., 2013; Wang et al., 2023). Some scholars have also explored the spatiotemporal evolution characteristics (Ma et al., 2016), AP enrichment effects, and potential ecological risk assessment of farmland utilization and have achieved fruitful results (Chen et al., 2022; Schoumans et al., 2015; Reijneveld et al., 2010). Most studies show that meteorological and topographic variables are the most important influencing factors on the spatial distribution of soil available phosphorus content (Hua et al., 2020; Miller et al., 2001; Cao et al., 2022). In different regions, the spatial variation of available phosphorus is closely related to soil properties and planting systems (Cao et al., 2012; Chad and James, 2019). Previous research mainly focused on certain administrative regions, crops, soil types, and land use types. There is still limited research on the spatial variation characteristics and influencing factors of soil AP in the cultivated layers of the water source area of the South-to-North Water Diversion Project (Tan et al., 2021; Wu, 2024). Previous studies have focused on describing or qualitatively analyzing the spatial differentiation of soil AP, often neglecting the exploration of interactions and the degree of influence among various factors. There has been a lack of quantitative analysis regarding these influencing factors and their interactions. Geodetectors are new statistical methods used to detect the spatial heterogeneity of events and reveal the driving factors behind them. These methods address the shortcomings of traditional approaches and provide a more comprehensive understanding of how influencing factors explain the spatial differentiation of soil nutrients.

The mid-route of the South-to-North Diversion Project (MSDP) is a strategic cross-basin water transfer project that alleviates the severe shortage of water resources in northern China (Yu et al., 2021). The water source area bears the arduous task of supplying water to the South-to-North Diversion Project, and water quality safety issues determine the success or failure of the entire project. The proportion of the agricultural population in this region is relatively high, and the vast rural areas are relatively backward, making it highly susceptible to ecological negative impacts caused by improper human development activities. It is a typical ecologically sensitive area, and the protection of the ecological environment in this region is the foundation and key measure to ensure good water quality. Based on this approach, the study uses Shiyan City, a key water source area of the MSDP, as a case area. It employs geostatistical methods to explore the spatial distribution characteristics of AP in the cultivated layers. By integrating geodetectors, the study investigates the main control factors and their interactions, aiming to reveal their inherent patterns and driving forces. The findings provide a theoretical basis for soil AP regulation and the improvement of farmland quality in the research area.

## 2 Materials and methods

### 2.1 Study area

**Figure 1:**
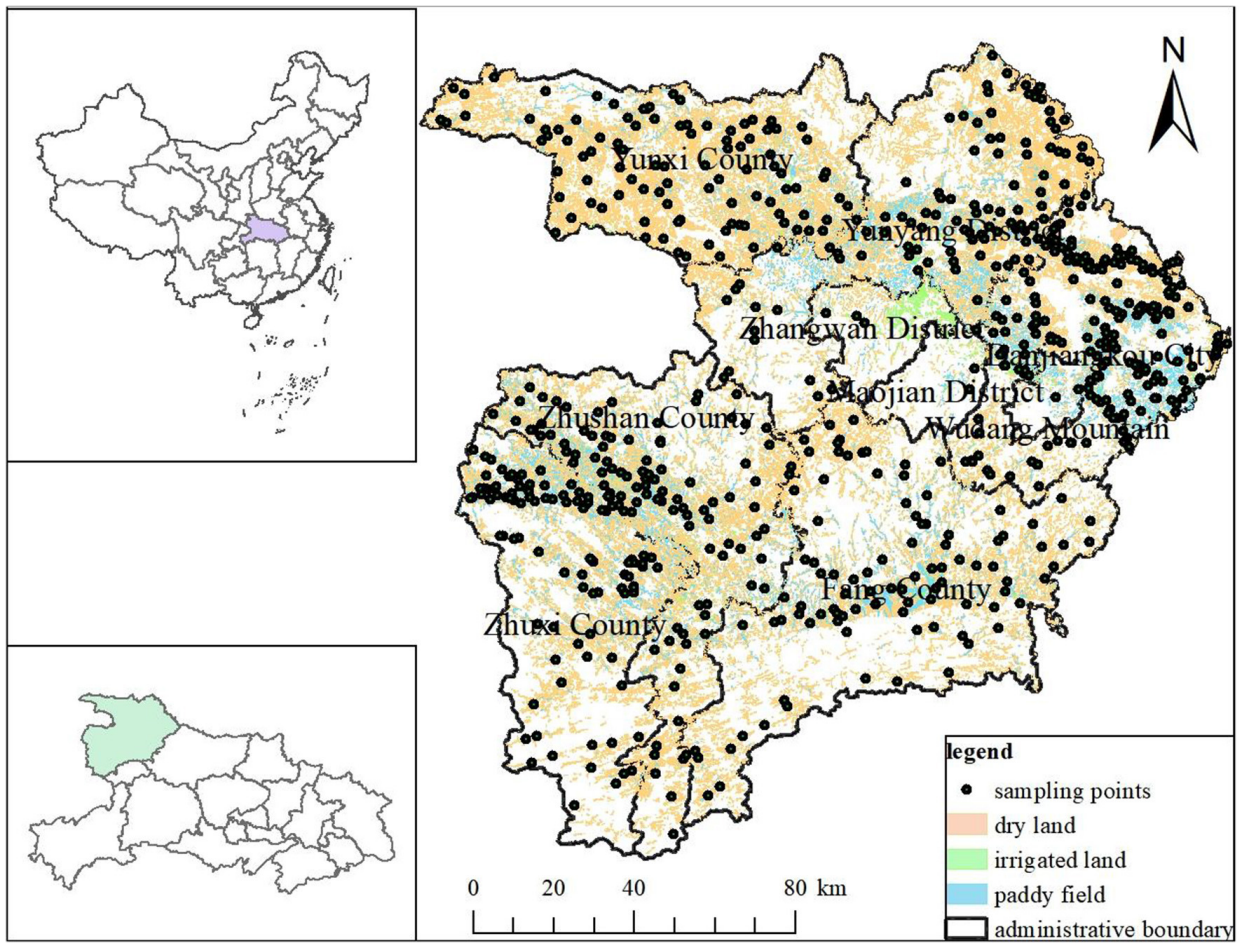
Distribution of sampling sites in the study area.

Shiyan City is located in the northwest Hubei Province and is the core water source area of the MSDP (109°29′-111°16′E, 31°30–33°16′N) (Figure 1). It is known as the “Green Lung of Central China” and the “Water Well of North China,” with an area of 23,680 km^2^. This region has a northern subtropical continental monsoon climate, with an average annual temperature of 15.4°C, an average annual precipitation of 870 mm, 1,650 h of sunshine, and a frost-free period of 224 days. At an altitude of 83–2,571 m, there are over 2,000 rivers. The landform types are hills, low mountains, middle mountains, and high mountains, suitable for the growth of various water and drought crops. The farming types are mainly wheat, corn, and rice. The main planting systems include rotation, monoculture, and intercropping, and yellow-brown soil, lime soil, and paddy soil are the main soil types.

### 2.2 Data sources and preprocessing methods

#### 2.2.1 Soil sample data

The soil sample data were sourced from the farmland quality survey and evaluation project in Shiyan City (Figure 1). After the autumn harvest of crops in 2020, sampling points were arranged according to the utilization conditions of cultivated land in each county and city. Five soil samples were collected from the top 0–20 cm layer using a “star” or “S” shape, according to the actual situation of the plot (Fink et al., 2016). After collection, the samples were mixed, and 1 kg was kept for further analysis. After air drying, grinding, and sieving, the soil sample was subjected to a sodium bicarbonate extraction molybdenum antimony colorimetric method to determine soil AP, a potassium dichromate volumetric method to determine soil organic matter, and a 2.5:1 soil water ratio extraction pH meter method to determine soil pH (National Agricultural Technology Extension Service Center, 2006). During sampling, GPS positioning was used to obtain the geographical location and altitude of each sampling point, and over 40 types of environmental background information were recorded and investigated, including soil parent materials, soil types, crop rotations, and land use. At the same time, geographical coordinates and altitude were recorded for each sampling point. Finally, 701 representative sample points were selected for research (Figure 1).

#### 2.2.2 Impact factor data

Based on existing research, the impact of soil AP spatial variation is mainly concentrated in eight aspects: terrain (Liu et al., 2022, 2016; Hua et al., 2020; Li et al., 2016), climate (Liu et al., 2022), soil type (Liu et al., 2022; Khan et al., 2018; Ma et al., 2016), soil pH (Liu et al., 2022; Zhao et al., 2011), soil organic matter (Khan et al., 2018), soil parent materials (Liu et al., 2022; Hua et al., 2020), land use (Liu et al., 2022; Khan et al., 2018; Hua et al., 2020), and soil management (Liu et al., 2022; Bieluczyk et al., 2024; Khan et al., 2018; Holger et al., 2018). Based on existing achievements, this research selected the following influencing factors:

- Structural factors: elevation (Elev), slope (Slope), mean annual temperature (Mat), mean annual precipitation (Map), soil type (Soil type), soil pH (Soil pH), and soil organic matter (SOM).
- Randomness factors: land use and planting system.

The elevation and slope data were calculated using ArcGIS 10.7 using digital elevation data with a horizontal resolution of 30 on the geospatial cloud platform. Climate data were obtained from the Resource and Environmental Science and Data Center, with a resolution of 500 m × 500 m. The data on soil type, land use status, and planting system were sourced from the land parcel survey.

#### 2.2.3 Data preprocessing

All vector data and raster data were converted to a unified projection coordinate system. According to the requirements of geographical exploration input variables, the land use data were categorized. Continuous data, including elevation, slope, temperature, and precipitation were classified into seven categories using the natural breakpoint method. The semi-variance function of soil AP was fitted using GS+9.0 software.

### 2.3 Research methods

#### 2.3.1 Geostatistical methods

The semi-variance function is the theoretical basis of geostatistics and is used in this study to reflect the spatial variation and correlation degree of the regionalized variable AP in the study area. The calculation formula is as follows:


$$\begin{array}{l} r\left(h\right)=\frac{1}{2N\left(h\right)}\overset{n}{\underset{i=1}{\sum }}{\left[z\left(x_{i}\right)-Z\left(x_{i}+i\right)\right]}^{2} \end{array}$$

#### 2.3.2 Geodetectors

Geodetectors are new statistical methods that detect spatial differentiation of geographical phenomena and reveal their underlying driving forces. They include several key factors: factor detection, interaction detection, risk detection, and ecological detection (Chad and James, 2019). Among them, factor detection uses a *q*-value to measure the explanation of factor *X* for the spatial differentiation of attribute *Y*, with a range of *q*-values of [0, 1]. The bigger the *q*-value, the stronger the explanatory power of the independent variable *X* for attribute *Y*, and vice versa.

The interaction detector is used to measure whether the interaction between two influencing factors will increase or decrease the explanatory power of soil AP spatial variation. If the *q*-value is closer to 1, it indicates that the interaction between the two factors is more significant (Table 1). Based on the results of factor detection and interaction detection, this article identified the dominant factors and dual-factor interaction results that affect the spatial variation of soil AP in Shiyan City.

**Table 1:** Types of interaction detection.

**Judgment criteria**	**Interaction results**
q(X_i_∩X_j_) < Min(q(X_i_)), (q(X_j_))	Non-linear attenuation
Min(q(X_i_)), (q(X_j_)) < q(X_i_∩X_j_) < Max(q(X_i_)), (q(X_j_))	Single-factor non-linear attenuation
q(X_i_∩X_j_) > Max(q(X_i_)), (q(X_j_))	Dual-factor enhancement
q(X_i_∩X_j_) = (q(X_i_)) + q(X_j_)	Independence
q(X_i_∩X_j_) > (q(X_i_)) + q(X_j_)	Non-linear enhancement

## 3 Results and analysis

### 3.1 AP descriptive statistical analysis

According to the soil AP classification method in the second soil survey, the soil AP in the study area was classified (Li et al., 2016) (Table 2), with a sample size of 5.99%, 14.27%, 25.11%, 34.52%, 14.27%, and 5.85% for levels I–VI, respectively. The AP content in the study area is concentrated in two moderate levels, accounting for 59.63% of the total. Table 3 shows that the AP content of 701 sample points in the study area ranges from 0.30 to 146.00 mg/kg, with an average value of 14.28 mg/kg. According to the AP classification of the second soil survey, the overall AP is in a moderate state, with a standard deviation of 14.96 mg/kg, reflecting the heterogeneity of the sample data. The coefficient of variation of AP is 104.76%, belonging to a strong degree of variation. The AP content changes greatly, with many extreme values and a relatively scattered distribution. In the process of soil management, targeted fertilization plans should be formulated according to local conditions and cannot be generalized.

**Table 2:** Classification standard of soil available phosphorous and frequency distribution of each class.

**Grade**	**Range (mg/kg)**	**Sample size**	**Ratio**
I (extremely rich)	>40	42	5.99%
II (rich)	20–40	100	14.27%
III (upper-middle)	10–20	176	25.11%
IV (middle-lower)	5–10	242	34.52%
V (less lacking)	3–5	100	14.27%
VI (extremely lacking)	< 3	41	5.85%

**Table 3:** Descriptive statistic of soil available phosphorous.

**Soil property**	**Sample size**	**Minimum**	**Maximum**	**Mean**	**Median**	**Standard deviation**	**Skewness**	**Kurtosis**	**Coefficient of variation (%)**
AP	701	0.30	146.00	14.28	9.24	14.96	0.09	15.58	104.76%

### 3.2 Analysis of spatial variation structure characteristics of AP

The semi-variance function of soil AP in the study area was fitted (Table 4). The optimal model was selected based on the following criteria: maximizing the coefficient of determination (*R*^2^) to approach 1, minimizing the residual sum of squares (RSS) to approach 0, and prioritizing the RSS value. The results show that the exponential model has the best fitting effect and can better reflect the good spatial structure of soil AP.

**Table 4:** Semivariogram model and its parameters of soil available phosphorous.

**Theoretic models**	**Nugget (C_0_)**	**Sill (C_0_ + C)**	**Nugget/sill (C_0_/C_0_ + C)**	**Range/m**	***R* ^2^**	**RSS**
Spherical	0.001	0.675	0.001	8,600	0.296	0.0834
Exponential	0.430	0.961	0.447	82,000	0.955	7.561E-03
Gaussian	0.079	0.675	0.117	7,447	0.299	0.0832
Linear	0.517	0.797	0.649	102,458	0.874	0.0149

Spatial variation mainly includes two parts: random variation and structural variation. In Table 3, C0/(C0 + C) is referred to as the block gold coefficient, which represents the degree of spatial heterogeneity. A high ratio indicates a significant degree of spatial variation caused by random parts (Xiaolan et al., 2007). On the contrary, a higher degree of spatial variation due to spatial autocorrelation is observed. It is generally believed that variables smaller than 0.25 have strong spatial autocorrelation. Variables with moderate spatial autocorrelation have coefficients between 0.25 and 0.75. Variables above 0.75 have weak spatial autocorrelation (Cambardella et al., 1994). For such variables, the variation is mainly random, which is not suitable for using spatial interpolation methods for prediction (Goovaerts, 1999). The lump gold value (C0) of soil AP is 0.430, indicating the presence of random factors causing variation at the current sampling density. The nugget coefficient is 0.447, showing a moderate degree of spatial autocorrelation, indicating that the spatial variation of soil AP in the study area is influenced by both structural and random factors. The range of the study area is 82,000 m, with a step size of 7,050.29 m, indicating that the sampling spacing set up in the study area is smaller than the range of soil AP, which can meet the needs of spatial variability evaluation in the study area.

### 3.3 AP Kriging interpolation mapping and analysis

**Figure 2:**
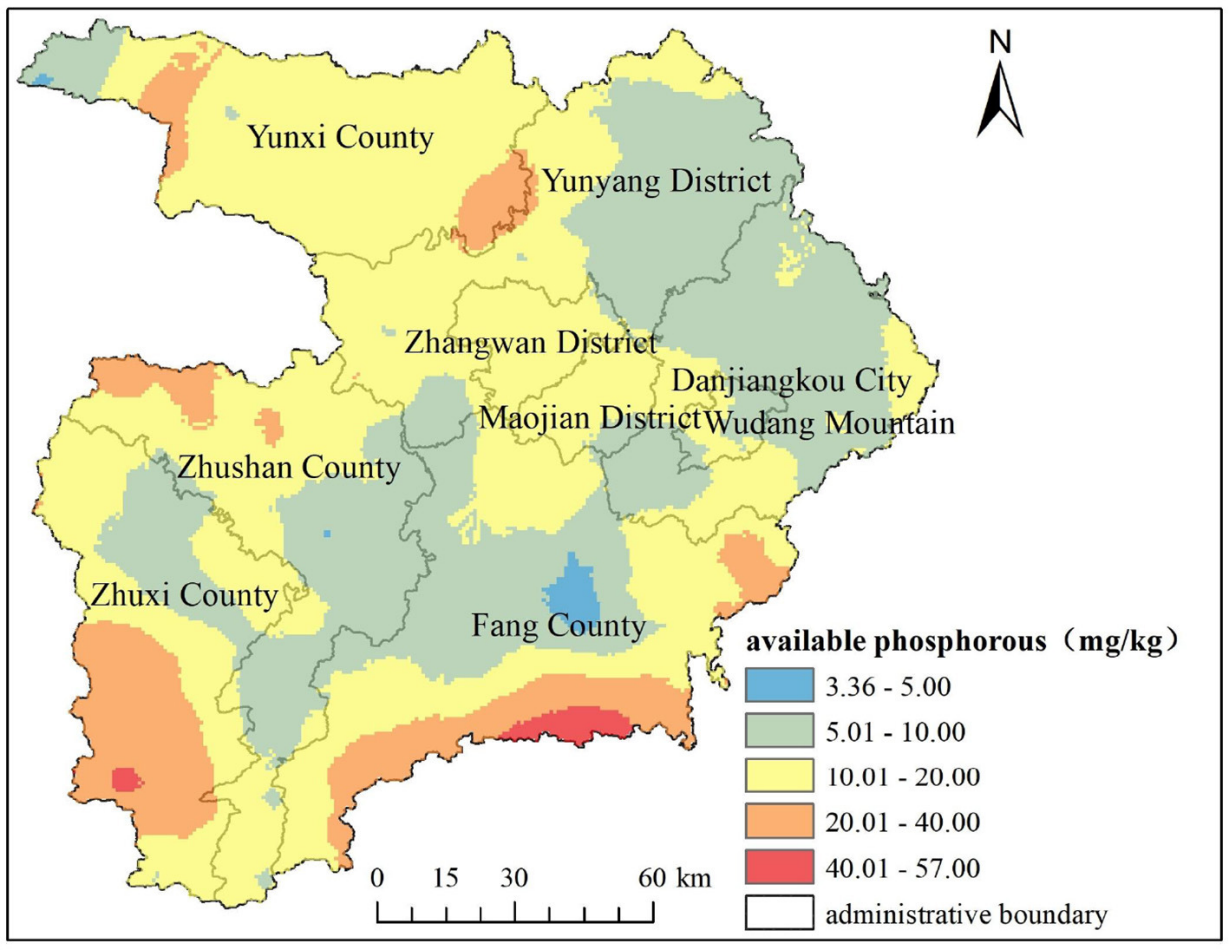
Spatial distribution of soil available phosphorous in the study area.

**Figure 3:**
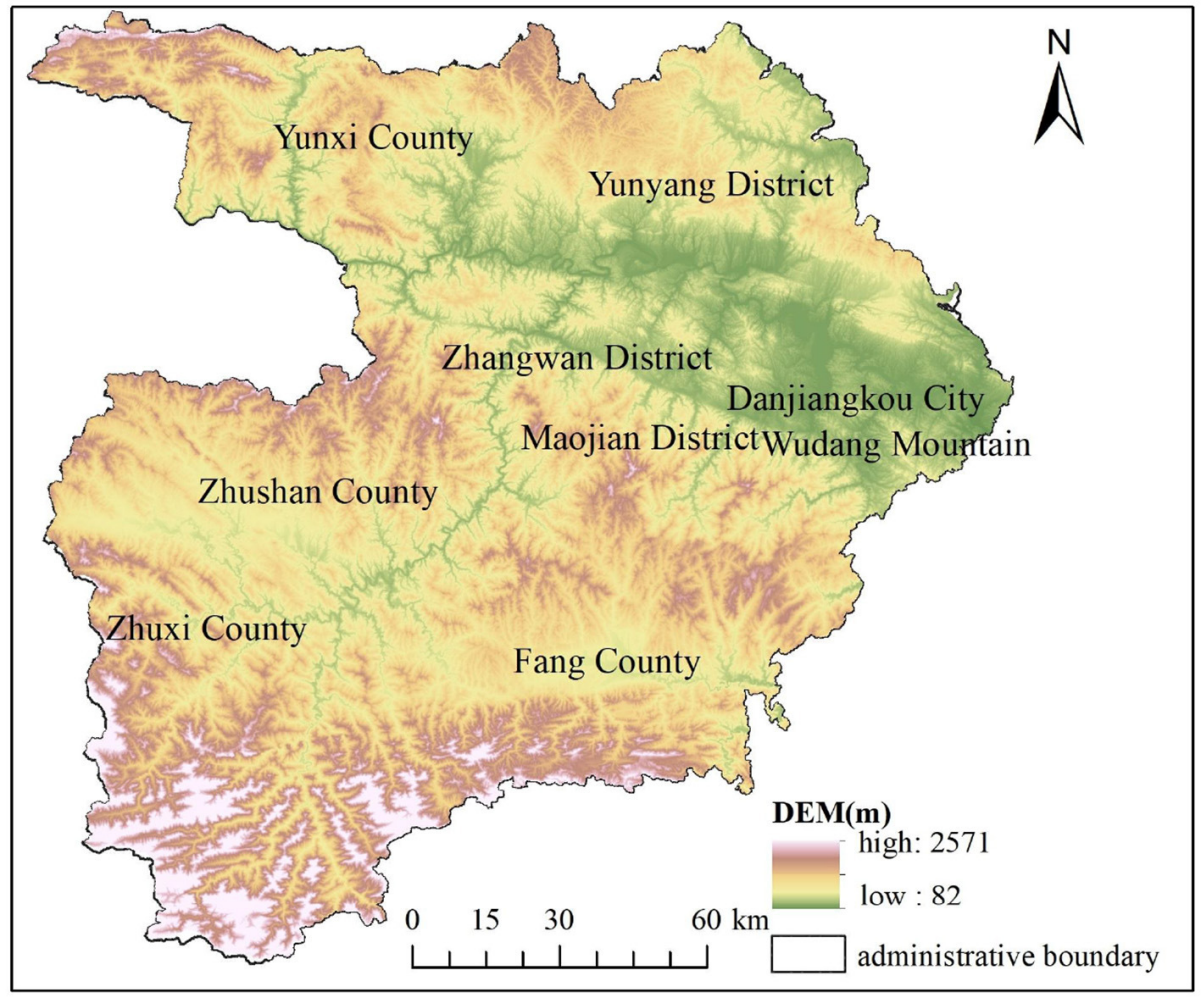
Elevation map of the research area.

The optimal parameters of Kriging interpolation were simulated using GS^+^9.0 software and then input into ArcGIS 10.7. The spatial distribution map of soil AP was created using ordinary Kriging interpolation. Figure 2 shows that the distribution area of extremely rich grade areas is relatively small, mainly distributed in the southwest of Zhuxichuan County and the southeast of Fang County, with a relatively high terrain; The lack of hierarchy is mainly distributed in the faulted basin centered around the Malan River Valley in the northern part of Fang County, where the granaries are located and the terrain is relatively low. The spatial distribution of soil AP shows an increasing trend from the Central Valley lowlands to the surrounding mountainous hills, which is strongly consistent with the terrain changes in the study area (Figure 3). According to the soil AP grading standards in the second soil survey, the soil AP content in the vast majority of the study area is mainly at levels III and IV, indicating that the average level of soil AP content in the study area is moderate and can meet the requirements of crop growth.

### 3.4 Analysis of geodetectors

#### 3.4.1 Factor detection analysis

The factor detector reflects the influence of geographical environmental factors on the spatial distribution pattern of soil AP and is measured by the magnitude of the *q*-value. The results show (Table 5) that the *q*-values of each influencing factor are arranged in descending order as elevation (0.1459), soil type (0.0707), planting system (0.0622), annual precipitation (0.0612), organic matter (0.0623), annual average temperature (0.0456), soil pH (0.0301), slope (0.0264), and farmland use (0.0104). Among them, the q-value of elevation is bigger than 0.1 and has the strongest explanatory power through a 1% significance test, which is the main controlling factor determining the spatial distribution pattern of AP in Shiyan City. The following factors are soil types, planting systems, annual precipitation, and organic matter. The *q*-values of other factors are relatively small and have weak explanatory power, which are secondary factors affecting the spatial heterogeneity of soil AP. Comparing the explanatory power of structural factors and random factors, it can be found that the *q*-values of structural factors such as terrain and climate factors are slightly higher than those of random factors such as planting systems and land use, indicating that structural factors have a relatively large driving force on the spatial variation of AP in cultivated land in Shiyan City, which is consistent with previous analysis.

**Table 5:** Geographical detection of the factors affecting the soil available phosphorous spatial variation.

**Factor type**	**Factor detects *q*-value**	**Interactive detection** ***q*****-value**								
			**X2**	**X3**	**X4**	**X5**	**X6**	**X7**	**X8**	**X9**
X_1_	0.1459^*^	0.1459								
X_2_	0.0264^*^	0.3224^#^	0.0264							
X_3_	0.0456^*^	0.2042^#^	0.1299^#^	0.0456						
X_4_	0.0612^*^	0.2301^#^	0.1440^#^	0.1425^#^	0.0612					
X_5_	0.0707^*^	0.2155	0.1943^#^	0.1346^#^	0.1537^#^	0.070				
X_6_	0.0301^*^	0.2604^#^	0.1012^#^	0.1361^#^	0.1756^#^	0.1637^#^	0.0301			
X_7_	0.0623^*^	0.3161^#^	0.1462^#^	0.1150^#^	0.1474^#^	0.1770^#^	0.1280^#^	0.0623		
X_8_	0.0104^***^	0.1605^#^	0.0501^#^	0.0724^#^	0.0884^#^	0.0888^#^	0.0605^#^	0.1028^#^	0.0104	
X_9_	0.0622^*^	0.2905^#^	0.2621^#^	0.1710^#^	0.2085^#^	0.1762^#^	0.1840^#^	0.2274^#^	0.0916^#^	0.0622

X1, X2, X3, X4, X5, X6, X7, X8, and X9, respectively, represent elevation, slope, mean annual temperature, mean annual precipitation, soil type, soil pH, soil organic matter, land use, and planting system; ^***^ and ^*^ significant at the 1% and 10% levels, respectively. ^#^Indicating non-linear enhancement.

#### 3.4.2 Interaction detection analysis

This study used interaction detectors to analyze the degree of interaction between nine factors and the spatial distribution of soil AP content in the study area (Table 5). In terms of dual-factor interaction, both factors exhibit non-linear enhancement or dual-factor enhancement effects. The maximum interaction factor is elevation slope (0.3224), followed by elevation organic matter (0.3161), elevation planting system (0.2905), slope planting system (0.2621), and elevation soil pH (0.2604). The explanatory power of the interaction is bigger than 0.25. In terms of comprehensive interaction, the strongest is elevation synthesis (total 1.9997), followed by planting system synthesis (1.6114), slope synthesis (1.3502), annual precipitation (1.2904), organic matter (1.1830), soil type (1.1502), annual average temperature (1.1057), soil pH (1.0459), and farmland utilization (0.7151). Overall, the interaction between elevation, planting system, and slope with other factors is quite prominent, which means that areas with significant differences in elevation, planting system, and slope often have significant differences in AP. The distribution of other factors will enhance the impact of elevation, planting system, and slope on the spatial distribution of AP in the cultivated layers.

## 4 Discussion

To further investigate the impact of various environmental variables on the differentiation of soil AP in cultivated land in the study area, a correlation analysis was conducted between soil AP and environmental variables (Table 6). It was found that soil AP was negatively correlated with altitude, field slope, annual precipitation, and soil organic matter content in the study area and negatively correlated with annual average temperature and soil pH.

**Table 6:** Correlation analysis of soil available phosphorous and environmental variables.

**Environmental variables**	**pH**	**SOM**	**Map**	**Mat**	**Elev**	**Slope**
Pearson	−0.149^**^	0.235^**^	0.179^**^	−0.166^**^	0.339^**^	0.089^*^
*P*-value	0.000	0.000	0.000	0.000	0.000	0.018

^**^At the 0.01 level (double-tailed), the correlation is significant.

^*^At the 0.05 level (two-tailed), the correlation is significant.

### 4.1 The impact of terrain factors on AP variation

Terrain factors affect the available phosphorus content in soil by influencing water and thermal conditions and the redistribution of soil-forming materials (Li et al., 2016). Table 6 shows that the AP content in the study area is significantly positively correlated with altitude and slope. The low-value areas of AP in the research area are mainly distributed in low-terrain areas, such as the Han River and its tributaries, the Duhe River, and the Malan River valley. The terrain is relatively flat and suitable for crop cultivation, making it a concentrated production area for vegetables and grain crops. Compared to areas with higher elevations, the intensity of land development and utilization in this area is higher, resulting in significant interference from farming activities. Crops are densely planted, and the phosphorus carried away by crop growth is relatively high, resulting in lower available phosphorus content in the soil. The high-value areas are mainly distributed in the northern, central, and southern regions with high elevations and steep slopes. Due to the vertical zonality of the climate, agricultural production in this area exhibits strong seasonality and a low rate of multiple cropping. The transportation convenience is poor, and there is no comparative advantage in output level. In addition, to increase household income, a large number of young and middle-aged people in the region have migrated for work, resulting in a shortage of rural labor and leading to the abandonment of cultivated land. In Shiyan City, where the climate is characterized by favorable rainy and hot conditions, these factors facilitate self-restoration of the ecological environment of this abandoned cultivated land, resulting in a high AP content. In addition, in areas with higher elevations, the slope of sampling points is relatively gentle, making it easier for phosphorus to accumulate in the soil and increasing the available phosphorus content. Dong et al. (2016) studied the spatial distribution of available phosphorus in tea garden soil and found that the higher the terrain and the greater the slope, the higher the phosphorus content, and vice versa. Hua et al. (2020) found a positive correlation between soil AP and elevation through research, which is consistent with the results of this study. Zhao et al. (2011) found that terrain and landforms are the main structural factors affecting the spatial differentiation of soil AP, but they found a significant negative correlation between soil AP content and altitude and slope. Wang Y. H. et al. (2016) found through their research on the tobacco growing areas in northern Sichuan in southwest China that due to the loss of available phosphorus in high-altitude areas and the enrichment of available phosphorus in low-altitude areas, soil AP is negatively correlated with elevations. From this, it can be seen that the impact of terrain factors on AP is very complex, which may be caused by differences in location conditions and research scales of different research areas, and the specific reasons may need further analysis.

### 4.2 The impact of climate factors on AP variation

The impact of climate on soil AP is mainly reflected in two aspects: temperature and precipitation. Previous studies have shown that fluctuations in soil temperature and moisture caused by changes in temperature and precipitation affect the conversion of phosphorus components within the soil through non-biological factors such as soil pH, nutrient content, and moisture content, as well as biological factors such as soil microorganisms and vegetation types, thereby affecting the available phosphorus content in the soil (Santos et al., 2019; Wang et al., 2019; Wu et al., 2020; Mei et al., 2019). High temperatures and precipitation make phosphorus in the soil easily weathered and released (Lin et al., 2009). Due to the influence of terrain, for every 100 m increase in altitude in the study area, the average temperature decreases by 0.55°C, and precipitation increases with altitude, with an increase of 35 mm for every 100 m increase (Wu et al., 2021). It is generally believed that the annual precipitation mainly affects the spatial distribution of soil AP through soil leaching. The more annual precipitation there is, the stronger the soil leaching effect, leading to the loss of available phosphorus in the soil (Miller et al., 2001). Precipitation mainly changes soil moisture and soil aggregate structure, causing soil leaching and reducing soil phosphorus content. It can also affect the migration and transformation of phosphorus elements, as well as the composition and availability of soil phosphorus elements, by controlling the biochemical processes of organic phosphorus mineralization (Wang R. Z. et al., 2016). Lambers et al. (2006) believe that under higher moisture conditions, the diffusion rate of soil phosphorus increases with the increase of soil moisture, accelerating the rate of plant and microbial uptake of phosphorus, resulting in lower available phosphorus content in the soil.

The annual average temperature mainly indirectly affects the availability of soil phosphorus by affecting the weathering rate of phosphorus-containing minerals and the activity of microorganisms in the soil during the soil formation process, thereby affecting the content of soil available phosphorus (Cao et al., 2022). Warming promotes the increase of dissolved phosphorus in soil by affecting the phosphorus content of litter, causing it to adsorb and precipitate with calcium carbonate, fixing dissolved phosphorus in the soil surface, and increasing the effective phosphorus content of the soil (Siebers et al., 2017). Wang et al. (2022) found that an increase in temperature may lead to a decrease in acid phosphatase activity and microbial biomass phosphorus content in cultivated land, reducing microbial activity and their ability to retain phosphorus. However, in this study, the AP content in the study area was significantly positively correlated with annual precipitation and negatively correlated with annual average temperature (Table 6). The areas with higher AP content in the study area were distributed in the southwest of Zhuxichuan County and the southeast of Fang County, which show higher precipitation and lower temperatures. Cao et al. (2022) found that in hilly areas of south China, high values of soil available phosphorus are mainly distributed in areas with low annual precipitation and high annual average temperature, which is inconsistent with the results of this study. According to the study by Wu et al. (2021), the soil organic matter content in Shiyan City shows a continuous decreasing trend with the decrease of elevation, the status of available phosphorus in soil is closely related to the content of soil organic matter, and in agricultural practice, increasing the content of soil organic matter can increase the desorption of solid phosphorus, enhance phosphorus activity, and increase the content of available phosphorus (Shen et al., 2014; Fei et al., 2021). Perhaps due to the lower temperature and weak soil microbial activity in high-altitude areas, the decomposition of organic matter is slow, which is conducive to the accumulation of organic matter. There are more nutrient elements accumulated in the soil, and the content of soil organic matter is significantly positively correlated with the content of available phosphorus, resulting in a higher content of AP in the soil. It is also possible that other factors such as elevation have a bigger impact on the spatial variation of soil AP in the study area than climate factors, leading to the masking of the impact of climate factors.

### 4.3 The impact of soil type on AP variation

Different soils in the study area have a significant impact on soil AP content (*F* = 9.849, *P* < 0.05), with brown soil having the highest content (64.18 mg/kg) and purple soil having the lowest content (10.55 mg/kg). The coefficients of variation for paddy soil and tidal soil are 123.49% and 103.07%, respectively, indicating strong variation, while others show moderate variation. The brown soil in the research area is acidic brown soil, and the developed parent material is mainly weathered mudstone, with a slightly acidic soil (Table 7). As shown in Table 6, there is a negative correlation between soil pH and soil AP content in the study area. Soil pH affects soil phosphorus availability by affecting the adsorption and fixation of soil phosphorus (Fei et al., 2021). The lower the soil pH, the stronger the acidity, and the bigger the adsorption and fixation effect of phosphorus by iron and aluminum oxides. It exists in the form of phosphate, and phosphorus fertilizers used in agricultural management are also easily adsorbed and fixed in large quantities, thereby increasing the content of available phosphorus in the soil (Chad and James, 2019). The moisture soil contains abundant carbonates and iron aluminum oxides, with high clay content and strong adsorption of phosphorus. The terrain of purple soil is hilly and undulating, with strong soil erosion, making it easy to lose effective phosphorus. The development degree of this soil type is relatively low, and good soil ventilation makes it difficult to accumulate organic matter. In addition, the utilization intensity of this soil type is relatively low, and the application amount of phosphorus fertilizer is also relatively low, resulting in a significantly lower accumulation rate of effective phosphorus in purple soil than in other soils. The average content of available phosphorus in the soil of this study is ranked as follows: brown soil > fluvo-aquic soil > paddy soil > yellow-brown soil > calcareous soil > yellow cinnamon soil>purple soil. Wang Y. H. et al. (2016) studied the spatial variation characteristics of soil available phosphorus in the tobacco growing areas of northern Sichuan of China and found that the average content of available phosphorus in five soil types was as follows: paddy soil > purple soil > yellow-brown soil > new soil > yellow soil. In both regions, the AP content of paddy soil is relatively high, but the AP content of yellow-brown soil in the study area is higher than that of purple soil, while the AP content of purple soil in northern Sichuan is higher than that of yellow-brown soil. It can be seen that the content of soil AP in different regions is complex and has regional characteristics.

**Table 7:** Descriptive statistic characteristics of available phosphorous in different soil parent materials.

**Soil type**	**Sample size**	**Minimum/ (mg/kg)**	**Maximum/ (mg/kg)**	**Mean/ (mg/kg)**	**Standard deviation**	**Coefficient of variation /(%)**
Fluvo-aquic soil	12	4.10	55.10	16.31	16.81	103.07
Yellow cinnamon soil	30	2.50	40.80	12.20	8.89	72.90
Yellow-brown Soil	454	0.74	98.80	14.15	13.46	95.15
Calcareous soil	36	2.10	39.20	12.41	10.60	85.36
Paddy soil	119	0.30	146.00	15.46	19.09	123.49
Purple soil	46	3.10	52.67	10.55	9.24	87.58
Brown soil	4	5.50	105.40	64.18	49.26	76.76

### 4.4 The impact of the planting system on AP variation

Due to the wide area, complex terrain, significant local climate differences, and complex planting system of Shiyan City, water and drought crops in the region are planted in rotation, intercropping, and monoculture. This complex planting system affects the availability of phosphorus in the soil through factors such as land use intensity, management measures, and crop growth habits. The cultivation system significantly affects the soil's available phosphorus content, and there are significant differences in AP content among different planting systems in the study area (*P* < 0.05, *F* = 9.849). As shown in Table 8, the highest AP content is in vegetable cultivation (27.06 mg/kg), and the lowest is in rice cultivation (9.89 mg/kg). From the perspective of coefficient of variation, the coefficient of variation of AP content under different planting systems ranges from 75.30 to 147.88%, all showing moderate degree of variation. The high AP content in vegetable cultivation is due to the high replanting index and good economic benefits of vegetable cultivation. The local farmers cultivate diligently, actively apply farm manure, and invest in more production materials. According to a questionnaire survey, a large amount of phosphorus fertilizer was input in the local vegetable planting season [with an average annual phosphorus fertilizer input of 6.05 kg/hm^2^ (calculated as P_2_O_5_)], resulting in a higher rate of phosphorus accumulation. In addition, to improve economic benefits, local governments introduced policies that benefit farmers and actively promoted the implementation of organic fertilizers instead of chemical fertilizers to improve vegetable quality. On the one hand, organic fertilizers themselves contain a large amount of available phosphorus. On the other hand, the mineralization and decomposition of organic matter release available phosphorus. The increase in organic matter content promotes the release of adsorbed phosphorus from iron and aluminum oxides in the soil due to competition, increasing the content of available phosphorus in the soil (Fink et al., 2016). Rice monoculture results in lower AP uptake during crop harvest and higher soil AP content. However, among all planting methods, rice monoculture has the lowest AP content, which may be due to long-term flooding of the rice field, resulting in the loss or transformation of available phosphorus in the soil. Wheat corn rotation, corn rice rotation, and rapeseed corn rotation may be due to the lower economic benefits of grain crops and the more extensive cultivation and management practices employed by farmers. On the other hand, crops may deplete more soil nutrients more rapidly than they are replenished, leading to nutrient imbalances in the cultivated land. Although the amount of straw returned to the field is relatively large and contributes to improving the AP content in the soil, the relatively low phosphorus content in straw leads to a lower AP content. In future agricultural production, it is necessary to adjust the cultivation methods and fertilization measures in a timely manner according to the different planting systems and spatial distribution characteristics of soil nutrients in the study area and pay attention to soil fertilization management.

**Table 8:** Descriptive statistic characteristics of soil available phosphorous in different cropping systems.

**Cropping system**	**Sample size**	**Minimum/ (mg/kg)**	**Maximum/ (mg/kg)**	**Mean/ (mg/kg)**	**Standard deviation**	**Coefficient of variation (%)**
Tea and orchard planting	79	0.91	82.73	12.34	13.90	112.66
Vegetable planting	15	5.91	105.40	27.06	24.29	89.78
Rice monoculture	55	0.30	51.70	9.89	9.50	96.02
Rapeseed rice rotation	28	2.38	55.87	19.47	15.09	77.50
Wheat rice rotation	24	2.00	146.00	21.97	32.48	147.88
Wheat corn rotation	106	2.00	51.10	15.28	11.50	75.30
Corn rice rotation	43	3.60	40.00	12.05	9.45	78.41
Potato corn intercropping	71	1.25	71.77	16.02	16.40	102.39
Rapeseed corn rotation	193	0.74	66.20	11.17	10.33	92.49
Corn monoculture	87	2.36	104.40	18.22	19.07	104.69

### 4.5 The impact of interaction factors on AP spatial variation

The purpose of an interaction detector is to test whether the interaction between each factor increases, decreases, or is independent of the dependent variable, to better explain the driving mechanism. From the interaction results of influencing factors on the spatial differentiation of soil available potassium in the study area, it can be seen that the interaction of various environmental factors is bigger than their individual effects, and there is a non-linear or dual-factor enhancement between various environmental factors. Lin et al. (2023) used a geodetic instrument to analyze the spatial heterogeneity of pH values in cultivated land in Anhui Province and found that the interactions between various factors mainly manifested as non-linear enhancement and dual-factor enhancement. Liu et al. (2018) used a geographical detector to analyze the spatial differentiation and influencing factors of soil phosphorus loss in the Bailongjiang River Basin in Gansu Province, China, and found that the factors showed a synergistic enhancement effect on phosphorus loss, and the interaction relationship was a coexistence of non-linear enhancement and dual-factor enhancement, which is similar to the results of this study.

## 5 Conclusion

(1) The average AP content in the topsoil of the study area is 14.96 mg/kg, indicating a moderate level of phosphorus availability. The spatial distribution of AP content exhibits a notable degree of variability. The spatial structure is well-fitted using an exponential model, and the AP content indicates a moderate degree of spatial autocorrelation. Structural and random factors jointly affect the spatial variation of AP. The AP enrichment of the cultivated soil is mainly distributed in the southwest of Zhuxichuan County and the southeast of Fangxian County, where the terrain is relatively high. The lack of hierarchy is mainly distributed in the faulted basins in the northern part of Fang County. The spatial distribution of soil AP shows an increasing trend from the Central Valley lowlands to the surrounding mountainous hills, which is strongly consistent with the terrain changes.
(2) The operation results of the factor detector show that the q-value descending order of the impact factors on the spatial heterogeneity of soil AP is elevation (0.1459), soil type (0.0707), planting system (0.0622), annual precipitation (0.0612), organic matter (0.0623), annual average temperature (0.0456), soil pH (0.0301), slope (0.0264), and farmland use (0.0104). Elevation is the main controlling factor determining the spatial distribution pattern of soil AP in Shiyan City.
(3) The interaction detection results indicate that both factors exhibit non-linear enhancement or dual-factor enhancement effects, showing an increased influence when combined than when considered as single factors. The interaction between various influencing factors is bigger than their individual effects, and the synergistic effect of the two influencing factors will enhance the explanatory power of SOM spatial variation. The maximum interaction factor is elevationnslope (0.3224), followed by elevationnorganic matter (0.3161), elevationnplanting system (0.2905), slopenplanting system (0.2621), and elevationnsoil pH (0.2604), all of which have explanatory power >0.25. In terms of interactive comprehensive effects, elevation has the strongest interactive comprehensive effect (total 1.9997), followed by planting system comprehensive effect (1.6114), slope comprehensive effect (1.3502), annual precipitation (1.2904), organic matter (1.1830), soil type (1.1502), annual average temperature (1.1057), and soil pH (1.0459). The explanatory power of interactive comprehensive effects is > 1. Overall, the interaction between elevation, planting system, slope, and other factors is quite prominent. This means that areas with significant differences in elevation, planting system, and slope often have significant differences in AP. The distribution of other factors will enhance the influence of elevation, planting system, and slope on the spatial distribution of AP in the plow layers. This study provides a scientific reference for maintaining ecological security in the water source area of the MSDP, improving the precise management of AP, and enhancing the quality of cultivated land.

## Data Availability

The original contributions presented in the study are included in the article/supplementary material, further inquiries can be directed to the corresponding author.
